# Metagenomic insights into urolithin formation from rambutan rind extract by rat faecal-derived microbiome

**DOI:** 10.1007/s00253-026-13841-x

**Published:** 2026-04-27

**Authors:** Wai-Kit Tow, Cindy Shuan Ju Teh, Chien Wei Ooi, Ronald F. S. Lee, Maalini Krishnasamy, Uma Devi Palanisamy, Usha Sundralingam

**Affiliations:** 1 School of Pharmacy, Monash University Malaysia, Jalan Lagoon Selatan, Subang Jaya, Selangor 47500 Malaysia; 2 Department of Medical Microbiology, Faculty of Medicine, Universiti Malaya, Kuala Lumpur, Malaysia; 3 Jeffrey Cheah School of Medicine and Health Sciences, Monash University Malaysia, Jalan Lagoon Selatan, Subang Jaya, Selangor 47500 Malaysia; 4 Department of Chemical Engineering, School of Engineering, Monash University Malaysia, Jalan Lagoon Selatan, Subang Jaya, Selangor 47500 Malaysia; 5 Thomson Hospital, Kota Damansara, Petaling Jaya, Selangor 47810 Malaysia

**Keywords:** Ellagic acid, Urolithin, Fruit by-products, Metagenomic, Anaerobic fermentation

## Abstract

**Abstract:**

Ellagitannins and ellagic acid are microbially converted into urolithins, metabolites associated with antioxidant, anti-inflammatory, and mitochondrial-related activities. Although several human-derived urolithin-producing strains and their associated enzymes have recently been characterised, the diversity of microbial strategies across host systems remains poorly understood. This study investigated urolithin production in the Sprague-Dawley rat faecal-derived microbial communities supplemented with rambutan rind extract, an ellagitannin-rich agricultural by-product containing 35–40% geraniin. Rambutan rind extract supplementation was associated with reduced isobutyric acid levels at study endpoint. Ex vivo anaerobic fermentation of hydrolysed rambutan rind extract (113 µM ellagic acid equivalent) resulted in the formation of urolithin C (9.4 ± 0.6 µM) and Isourolithin A (12.5 ± 0.6 µM) by day 9. Shotgun metagenomics analysis revealed very low relative abundance of *Actinobacteria* (< 0.009%), despite this phylum encompassing most previously characterised urolithin-producing taxa. Canonical ellagic acid degradation genes and the MetaCyc EA degradation pathway were not detected. Comparative pathway analysis indicated overlap in general metabolic pathways with *Ellagibacter isourolithinifaciens* DSM 104140^T^ reflecting shared metabolic frameworks rather than conserved urolithin biosynthetic pathways, with highly divergent homologues (Eadh1, Eadh2, Eadh3, and Ucdh). Together, these findings demonstrate that rambutan rind extract can support urolithin formation in rat faecal-derived microbial consortia and highlight functional associations consistent with alternative or yet-uncharacterised microbial strategies for ellagitannin biotransformation. These findings support a discovery-driven framework for investigating urolithin biotransformation in non-human gut microbiomes using ellagitannin-rich agricultural substrates.

**Key points:**

• *Rambutan rind extract supports urolithin formation in rat-derived gut microbiota.*

• *Substrate concentration influences urolithin production under ex vivo conditions.*

• *Rat gut microbiota shows homologues’ divergence in urolithin-associated proteins.*

**Supplementary Information:**

The online version contains supplementary material available at 10.1007/s00253-026-13841-x.

## Introduction

Dietary ellagitannins (ET) and ellagic acid (EA), abundant in fruits such as pomegranate, berries, and nuts, undergo extensive microbial transformation in the gut to yield urolithins (Uro) (Zhang et al. 2023b). This class of metabolites exhibit diverse bioactivities, including antioxidant and anti-inflammatory activities, as well as the promotion of mitochondrial biogenesis (Tow et al. 2022). Increasing evidence suggests that many of the health benefits attributed to ET-rich foods are mediated not by the parent compounds themselves, but by these microbiome-derived metabolites. However, Uro production is highly variable across individuals and populations, reflecting differences in gut microbial composition and functional capacity. Despite growing interest in Uro, the microbial taxa and enzymatic frameworks underpinning their biosynthesis remain incompletely characterised.

To date, only a limited number of bacterial strains have been identified as capable of converting EA into Uro, including *Gordonibacter* spp. (Kim et al. 2024; Selma et al. 2014), *Enterocloster* spp. (Iglesias-Aguirre et al. 2023), *Lactobacillus plantarum* CCFM1286, CCFM1290, CCFM1291 (Zhang et al. 2023a), *Enterococcus faecium* FUA027 (Zhang et al. 2022), *Bifidobacterium pseudocatenulatum* INIA P815 (Gaya et al. 2018), *Streptococcus thermophilus* FUA329 (Liu et al. 2022), and *Ellagibacter isourolithinifaciens* DSM 104140^T^ (Selma et al. 2017). These organisms represent a narrow subset of the gut microbiome, and most known Uro-producing strains have been isolated from human hosts. Consequently, Uro biotransformation in non-human gut ecosystems remains poorly explored, and it is unclear whether similar or alternative functional strategies exist across different host species. Addressing this gap is important not only for mechanistic understanding, but also for the development of preclinical models and sustainable bioprocessing strategies.

Rambutan (*Nephelium lappaceum* L.) rind is an abundant agricultural by-product in Southeast Asia and has been reported to contain exceptionally high levels of geraniin, a major ET accounting for approximately 35–40% of the crude extract (Palanisamy et al. 2011). Given its high ET content and limited direct bioavailability, rambutan rind extract (RRE) represents a promising, yet underexplored, agricultural by-product for valorisation as a substrate for microbial Uro production. By utilising fruit wastes, it addresses both environmental and industrial demands, reducing waste generation while promoting the circular use of natural resources to yield health-promoting metabolites. Previous studies have largely relied on purified EA or ET-rich foods of human dietary relevance, whereas the biotransformation potential of ET-rich fruit by-product streams has received comparatively little attention (Wu et al. 2025; Zhang et al. 2023a; Zhao et al. 2025).

Progress in elucidating Uro biosynthesis has been hindered by methodological constraints. Culture-based approaches are laborious and have identified only a limited number of Uro-producing isolates, while sequence-based gene annotation often fails to resolve the enzymes responsible for key steps such as dehydroxylation and ring cleavage. Many enzymes implicated in polyphenol metabolism remain hypothetical or poorly characterised, complicating the direct association of specific metabolic steps with microbial lineages. In this context, shotgun metagenomics provides a powerful, culture-independent framework to explore the functional potential of complex microbial consortia and to identify candidate pathways and metabolic signatures associated with Uro production, particularly in systems that fall outside well-characterised human microbiomes.

Rodent models provide a controlled experimental platform for investigating microbial polyphenol metabolism, allowing systematic evaluation of microbial biotransformation processes that may not be readily captured in heterogenous human populations. In this study, Sprague-Dawley rats were employed as a controlled non-human model to investigate the microbial conversion of rambutan rind extract (RRE) into Uro and to explore the functional landscape of the associated gut microbiota. By integrating in vivo supplementation with ex vivo fermentation, targeted metabolite profiling, and shotgun metagenomics, this work aimed to (i) evaluate the capacity of Sprague-Dawley rat gut microbiota to produce Uro from an ET-rich fruit by-product substrate, (ii) assess factors influencing conversion efficiency relative to EA, (iii) characterise taxonomic and functional features associated with Uro-producing microbial consortia, and (iv) evaluate the sequence similarity of predicted microbial proteins to previously characterised Uro-associated enzymes to explore potential conservation or divergence of the Uro biosynthetic pathway. Rather than defining definitive biosynthetic pathways, this study adopts a discovery-driven approach to identify functional associations and alternative metabolic strategies that may support Uro biotransformation beyond the human gut ecosystem.

## Materials and methods

### Experimental overview

This study comprised four integrated experimental components: (i) an in vivo dietary intervention in Sprague-Dawley rats to evaluate the safety and metabolic impact of RRE, (ii) ex vivo anaerobic fermentation of rat faecal microbiota to assess Uro production from ET-rich substrates, (iii) targeted chemical and metagenomic analyses to characterise microbial metabolites and functional potential, (iv) targeted BLAST against characterised Uro-producing genes. Faecal samples collected from control and RRE-supplemented rats were used for short-chain fatty acids (SCFAs) profiling and as inoculum for anaerobic fermentation assays. Uro production was quantified from fermentation broths using high-performance liquid chromatography (HPLC)-multiple wavelength detector (MWD), while shotgun metagenomic sequencing was performed on selected fermentation cultures to investigate taxonomic composition and predicted functional features associated with Uro-producing microbial consortia. This integrated workflow (Supplementary Fig. 1) enabled the evaluation of microbial biotransformation capacity and functional associations under controlled experimental conditions.

### Materials

RRE used for the broth supplementation was extracted using 95% ethanol (EtOH) (1:10 w/v) (Sigma-Aldrich, MO, USA). Acid hydrolysis was performed on RRE to obtain hydrolysed RRE using 37% (v/v) hydrochloric acid (HCl) sourced from Systerm (Selangor, Malaysia). All chemicals and reagents used for the extraction and chromatographic work were of analytical grade. Ultrapure water (HPLC-grade) was prepared using Milli-Q reverse osmosis system (Millipore, Bedford, USA) and met the European Pharmacopoeia standards. Eighty-five percent (v/v) formic acid (CH_2_O_2_) (Merck, Darmstadt, Germany) was used to supplement the mobile phase solvent and sample acidification. Acetonitrile (ACN) (Merck, Darmstadt, Germany) was used as the extraction solvent and mobile phase solvent. Methanol (MeOH) (Merck, Darmstadt, Germany) was used to re-dissolve the extracted dried samples. Working standards of Uro A, B, C, and IsoUro A (Sigma-Aldrich, MO, USA) used for the method development were of HPLC grade with a purity of ≥ 97%. ABB (CM0957) (16 g of peptone, 7 g of yeast extract, 5 g of sodium chloride, 1 g of starch, 1 g of dextrose, 1 g of sodium pyruvate, 1 g of arginine, 0.5 g of L-cysteine hydrochloride, 0.4 g of sodium bicarbonate, 0.5 g of ferric pyrophosphate, 1 g of dithiothreitol, 0.5 g of sodium thioglycolate, 0.005 g of haemin, and 0.0005 g of vitamin K, per litre, respectively) was purchased from Thermo Fisher Scientific, Inc. (Waltham, MA, USA).

### Animal study design

Five- to six-week-old male Sprague-Dawley rats were used and housed at the animal holding facility of Monash University Malaysia. A total of 10 rats were used for this study. Rats were acclimatised for 3 days prior to the study under a 12-h light/12-h dark, with ad libitum access to food and water, after which they were randomised into two groups of five animals each (*n* = 5). Briefly, (i) one group was fed on a standard certified laboratory rat chow as the control group with phosphate-buffered saline (PBS) as the vehicle, while (ii) the other group was orally gavaged daily with the RRE (115 mg/kg per body weight in PBS) (Moorthy et al. 2022). The animals were housed separately in clean cages and fed with the selected specimen(s) daily for 2 weeks. Prior to supplementation, the rats were fasted overnight from food and monitored for their condition. Their faecal matter was collected daily for 2 weeks in their respective sterilised stool collection containers and pooled together by group for analysis. The faeces were processed within 30 min or stored at −80 °C until further analysis.

### Rat euthanasia

On day 14, both groups of animals were humanely euthanised following intraperitoneal administration of ketamine (100 mg/kg BW) and xylazine hydrochloride (10 mg/kg BW). Once anaesthetised, exsanguination was performed via cardiac puncture using a 21G needle inserted into the left ventricle through the chest wall. Following exsanguination, the animals were dissected for organ collection (brain, liver, heart, kidney, and colon). All harvested organs were immediately fixed in 10% neutral-buffered formalin for at least 24 h in preparation for histological analysis.

### Histopathology observations

Small blocks of tissue were taken from the organs harvested and processed using an automated tissue processor (Leica TPC1020 Automatic Tissue Processor, Leica, Germany). The total processing time was 15.5 h. After processing, the tissues were sectioned to a thickness of 5 µm using a rotary microtome (Leica, Germany) and dried overnight in an oven at 37 °C. The sections were stained according to the Harris haematoxylin and eosin (H&E) staining protocol and examined under a light microscope at 10 × magnification for pathological changes.

### Substrate conversion comparison study

Briefly, 1 ml of faecal suspension was inoculated into a Schott bottle containing 100 ml of anaerobic basal broth (ABB) (Oxoid) supplemented with either 20 µM of EA (Sigma-Aldrich) or 20 µM EA equivalent (EAE) from RRE, respectively. The substrates were first dissolved in 0.1% of propylene glycol (PPG) before being added to the broth. After 24 h of anaerobic incubation at 37 °C under N_2_/H_2_/CO_2_ (80:10:10), 5 ml of samples was collected once daily from the culture (starting from day 1) for HPLC analysis.

### *Ex vivo* fermentation of hydrolysed rambutan rind extract using rat faecal microbiota

Briefly, 1 g of the faeces was diluted with 10 ml of anaerobic basal broth (ABB) (Oxoid) (1/10 w/v) and homogenised in a stomacher. Further dilution was performed using ABB to determine metabolic activity by mixing 1 ml of faecal suspension and 100 ml of ABB supplemented with 113 µM ellagic acid equivalent (EAE) of hydrolysed RRE (Sigma-Aldrich), incubated at 37 °C under anaerobic conditions (N_2_/H_2_/CO_2_ (80:10:10)) using an anaerobic chamber. Five millilitres of samples was collected once per day from the culture for HPLC analysis.

### HPLC-MWD analysis of urolithins

The broth samples were extracted with 5 ml of ACN:H_2_O:CH_2_O_2_ (80:19.9:0.1), vortexed for 2 min and centrifuged at 3500 g for 10 min. The extracted samples were evaporated, and the dry samples were then re-dissolved in 500 µl methanol. A 0.22-µm PTFE syringe filter membrane (Evergreen Engineering & Resources, Selangor, Malaysia) was used to filter the extract prior to injecting into HPLC-MWD for analysis.

Quantification of the Uro A, B, C, and isourolithin A (IsoUro A) was carried out using an Agilent 1260 Infinity II HPLC system equipped with a quaternary pump and degasser, coupled with a MWD G7165A (Agilent, Santa, Clara, CA), and a 100-µl multi-autosampler loop, along with Phenomenex Luna RP C18(2) column (250 × 4.6 mm, 5 µm) (Torrance, CA, USA). The data acquisition was performed using ChemStation OpenLab CDS software (C.01.10[287]). The optimised chromatographic parameters were based on a previously established HPLC-MWD method (Supplementary Table 1–3) (Tow et al. 2025). Briefly, detection was performed at 305 nm, with a flow rate of 1.0 ml/min and column temperature maintained at 25 °C. A representative chromatogram of the HPLC-MWD quantification method demonstrates effective separation between compounds without overlaps (*R*_s_ value ≥ 2) (Supplementary Fig. 2 and Supplementary Table 4). Comparison between solvent and sample matrix spiked with Uro (broth media inoculated with Sprague-Dawley rat’s faecal matter) revealed no statistically significant matrix effect (*p* > 0.05 for all compounds) (Supplementary Table 5).

### Faecal sample extraction for GC–MS analysis

Faecal samples were removed from −80 °C storage and allowed to thaw for 15 min. Samples were manually homogenised with a small spatula. One hundred milligrams of the sample was diluted with 1993 µl of diluent and an additional 7 µl of phosphoric acid (85% w/w). Samples were then vortexed for 3 min on a Vortex Genie 2 (Scientific Industries) to allow complete homogenisation. Samples were then centrifuged at 12,500 rpm for 10 min. The supernatant was further diluted to a final concentration of 10 mg/ml. A total of 23.3 µl of 7.9 mM internal standard (IS) 4-methyl valeric acid was added to each sample to a final concentration of 200 µM.

### GC–MS method conditions and pulsed splitless injection technique

The GCMS method conditions were performed to the exact conditions as described by Gray et al*.* (2021) (Gray et al. 2022). A 8890 Agilent Gas Chromatograph (model G3542A) with a 5977 C Mass Selective Detector (model G7077C) and 7693 A Automatic Liquid Sampler (model G4513A) was used for the quantification of SCFA.

Data analysis was performed on an Agilent MassHunter Unknown Analysis (Agilent, version 10.2). A Hydroguard Water-Resistant Guard Column (Restek, 5 m length, 0.25 mm ID) was used in combination with a high-polarity, DB-FATWAX Ultra Inert PEG column (Agilent J&W, 30 m length, 0.25 mm ID, 0.25 film thickness). A Vu2 Column Union (Restek, fits 0.18 to 0.28 mm ID columns) was used to connect the guard and analytical column in combination with a Deactivated Universal Press-Tight Connector (Restek). Fifteen percent graphite/85% Vespel Ferrules (Agilent, 0.4 mm ID) were used to connect the guard column to the inlet and the mass selective detector. Pulsed splitless injection mode was used to inject 0.5 µl of sample into the sample inlet lined with a single taper splitless liner with glass wool (Agilent 4 mm × 6.47 mm × 78.5 mm) at 250 °C. The injection pulse pressure was set to 25.0 psi (2.25 ml/min) for 0.55 min, while the purge flow to the split vent was set to 50.0 ml/min at 0.55 min. After 0.55 min, the helium gas (Alpha Gas Solution, purified helium, 99.999%) flow rate was set to a constant flow rate of 1.5 ml/min (16.7 psi), which was purified upstream with a Gas Clean Purification System (Agilent) filtering oxygen, moisture, and organic contaminants.

Other inlet consumables included an Advanced Green inlet Septum (Agilent, 11 mm), Fluorocarbon O-ring (Agilent), and Gold Seal with washer (Agilent) for the bottom of the inlet. The Advanced Green Septa were used in this method. The temperature ramp for the oven of the gas chromatograph started at 80 °C for 2.50 min and increased to 230 °C at a rate of 15 °C/min, then increased to 245 °C at a rate of 30 °C/min, and was held for 2 min. The total run time was 15 min, while the solvent delay on the detector was set at 4 min. The mass selective detector was operated in electron impact ionisation mode at an energy of 70 electron volts. The scanning mass range 40.00–150.00 m/z was selected. The identification of SCFAs was confirmed using the National Institute of Standards and Technology Library, 2020 (NIST20).

### DNA extraction and shotgun metagenomic sequencing

The DNA was extracted from the culture media inoculated with 20 µM EA (Experimental) or PBS (control) on day 7 of incubation corresponding to the first appearance of detectable Uro C and IsoUro A using the QIAGEN DNeasy® Blood & Tissue Kit (QIAGEN, Germany), according to the manufacturer’s protocol (Purification of total DNA from crude lysates using the DNeasy® Blood & Tissue Kit, 2023). DNA concentration and purity were assessed using a NanoDrop™ 2000 spectrophotometer (Thermo Fisher Scientific, USA), and DNA integrity was verified by agarose gel electrophoresis prior to sequencing (Supplementary Fig. 3).

Shotgun metagenomic libraries were prepared and sequenced on an Illumina NovaSeq platform with paired-end 150 bp (PE150). The minimum output and size per sample were 6.5 Gb at 14 million total reads (QIAGEN 2023). Metagenomic sequencing was performed to characterise the taxonomic composition and predicted functional potential of microbial consortia enriched during Uro-producing fermentation, rather than to directly infer in vivo gut microbial function.

### Bioinformatic processing and functional profiling

Raw sequencing reads were processed using the bioBakery workflows v4.0.0a1 (McIver et al. 2018). Quality filtering and host contamination removal were performed using KneadData (v0.12.3). Taxonomic profiling was conducted using MetaPhlAn (v4.2.2) to obtain species-level relative abundance estimates, while functional profiling was performed using HUMAnN (v4.0.0.alpha.1) to infer pathway and gene family abundance.

**Fig. 1:**
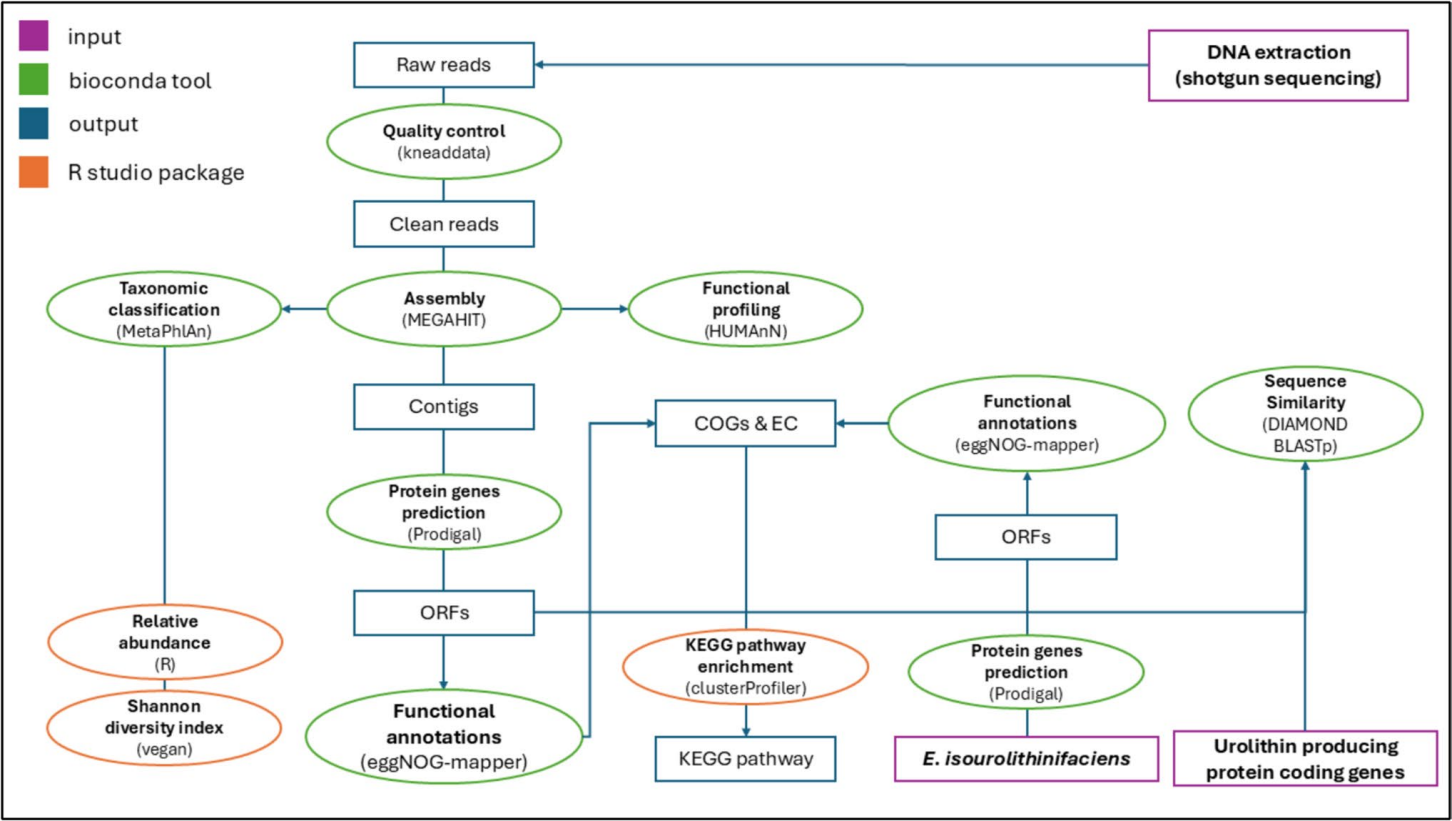
Metagenomics workflow for the taxonomic and functional profiling of the Uro-producing rat faecal-derived fermentation microbiota

Open reading frames were predicted from assembled contigs using Prodigal (v2.6.3), and functional annotation was carried out using eggNOG-mapper (v2.0.1) to assign Clusters of Orthologous Groups (COGs) and predicted enzyme functions. KEGG pathway enrichment analysis was performed using the clusterProfiler package in R (v4.16.0) to identify overrepresented metabolic pathways associated with Uro-producing fermentation cultures. Functional annotations were interpreted as indicators of predicted metabolic potential rather than confirmed enzymatic activity. The workflow is as illustrated in Fig. 1.

### Metagenome assembly and binning

Metagenome assembly was performed using MEGAHIT (v1.2.9), and read mapping rates back to assembled contigs were conducted using Bowtie2 (v2.5.4) to assess assembly quality. Metagenome-assembled genomes (MAGs) were generated using MetaBAT2 (v2.15), and MAG quality assessment and taxonomic classification were performed using GTDB-Tk (v2.4.1) with the GTDB reference database (release R226).

Predicted genes within MAGs were annotated using Prodigal and eggNOG-mapper to associate predicted functions with taxonomically assigned genome bins. This approach enabled the exploration of functional features associated with specific microbial taxa enriched during fermentation, while acknowledging that such associations do not establish causal roles in Uro biosynthesis.

### Comparative BLASTp analysis against *Ellagibacter isourolithinifaciens*

To explore potential functional similarities with known Uro-producing bacteria, the predicted protein-coding sequences from *E. isourolithinifaciens* DSM 104140^T^ were used as queries in BLASTp searches against the assembled metagenomic protein catalogue. The reference proteome of *E. isourolithinifaciens* DSM 104140^T^ was retrieved from the NCBI GenBank database. Predicted proteins from the metagenome were generated using Prodigal in metagenomic mode from MEGAHIT-assembled contigs and compiled into a FASTA database. A BLAST database was constructed using BLAST+ (v2.16.0), and the searches were performed using default scoring parameters and low-complexity filtering enabled.

Candidate matches were filtered using conservative thresholds (percentage identity > 30%, E-value ≤ 1e⁻^5^), and results were used to assess functional similarity at the pathway level rather than to infer the presence of conserved Uro biosynthetic enzymes. This comparative analysis was intended to provide contextual insight into shared metabolic frameworks rather than definitive evidence of homologous Uro biosynthesis pathways.

### DIAMOND BLASTp analysis against Eadh1, Eadh2, Eadh3, and Ucdh

To specifically assess whether the rat microbial consortium encodes homologues of recently characterised Uro biosynthesis enzymes, targeted homology searches were performed against key proteins reported by Bae et al. (2025). A curated reference protein database was manually assembled from the published sequences of experimentally validated enzymes involved in Uro biosynthesis (Supplementary Table 6) (Bae et al. 2025). Homology searches were conducted using DIAMOND BLASTp (v2.0.15) against the predicted protein sequences derived from the metagenomic assembly. Searches were performed using an *e*-value of ≤ 1e⁻^4^ and a minimum percentage identity of ≥ 20% parameters selected to allow detection of potentially distant homologues. Query coverage was calculated as the proportion of the query sequence length spanned by the alignment, expressed as a percentage. This targeted analysis was designed to determine whether the rat consortium encodes orthologues of the enzyme recently identified in human gut bacteria or whether Uro production in this system may involve phylogenetically distinct enzymes or alternative metabolic strategies.

### Statistical analysis

The statistical comparison of Uro production and pH changes between two substrates, EA and RRE, was performed using two-way repeated-measure ANOVA followed by Sidak’s multiple comparisons post hoc test to assess the differences between substrate supplementation across time points. Assumptions of normality and sphericity were verified prior to analysis. Statistical significance was determined using a two-tailed threshold of *p* < 0.05. The α-diversity indices were calculated using Shannon’s Diversity Index for taxonomic and metabolic pathway profiles. Bar plots were generated using the ggplot2 R package (v3.5.2). Shotgun metagenomic sequencing was performed on one sample per group (control and treatment). As such, statistical analyses were not applicable, and the results are reported descriptively, and the findings should be interpreted with caution. All statistical analyses were performed in R Studio (v2025.0.1, Build 513).

## Results

### Histological findings

Histopathological analysis of the brain, colon, liver, heart, and kidney, from Sprague-Dawley rats administered with RRE at a dosage of 115 mg/kg BW for 2 weeks revealed no significant histopathological abnormalities. All examined tissues demonstrated normal histoarchitecture, with no evidence of inflammation, necrosis, dysplasia, or malignancy, indicating that the administration of RRE did not induce any pathological changes compared to the control group (SupplementaryFig. 4). Across the organs examined, no treatment-associated pathological alterations were detected within the 2-week treatment period.

### Short-chain fatty acids profiling in rat faecal matter

**Fig. 2:**
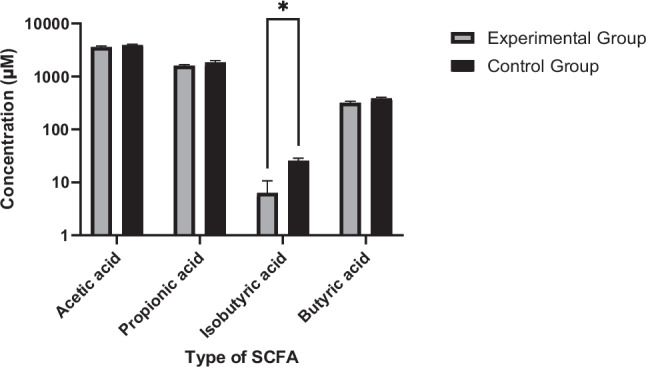
SCFA profiles in faecal samples of SD rats. Note: Data are presented as mean ± SD (*n* = 5 per group). Experimental group, fed with rambutan rind extract; the control group, fed with normal saline. *Statistically significant differences in isobutyric acid between experimental and control groups (*p* < 0.05)

Short-chain fatty acid (SCFA) analysis of faecal samples collected on day 14 revealed a statistically significant reduction in isobutyric acid levels in the RRE-fed group compared with the control group (*p* < 0.05) (Fig. 2). No statistically significant differences were observed for other measured SCFAs between groups.

### *Ex vivo* fermentation of hydrolysed rambutan rind extracts using rat faecal microbiota

**Fig. 3:**
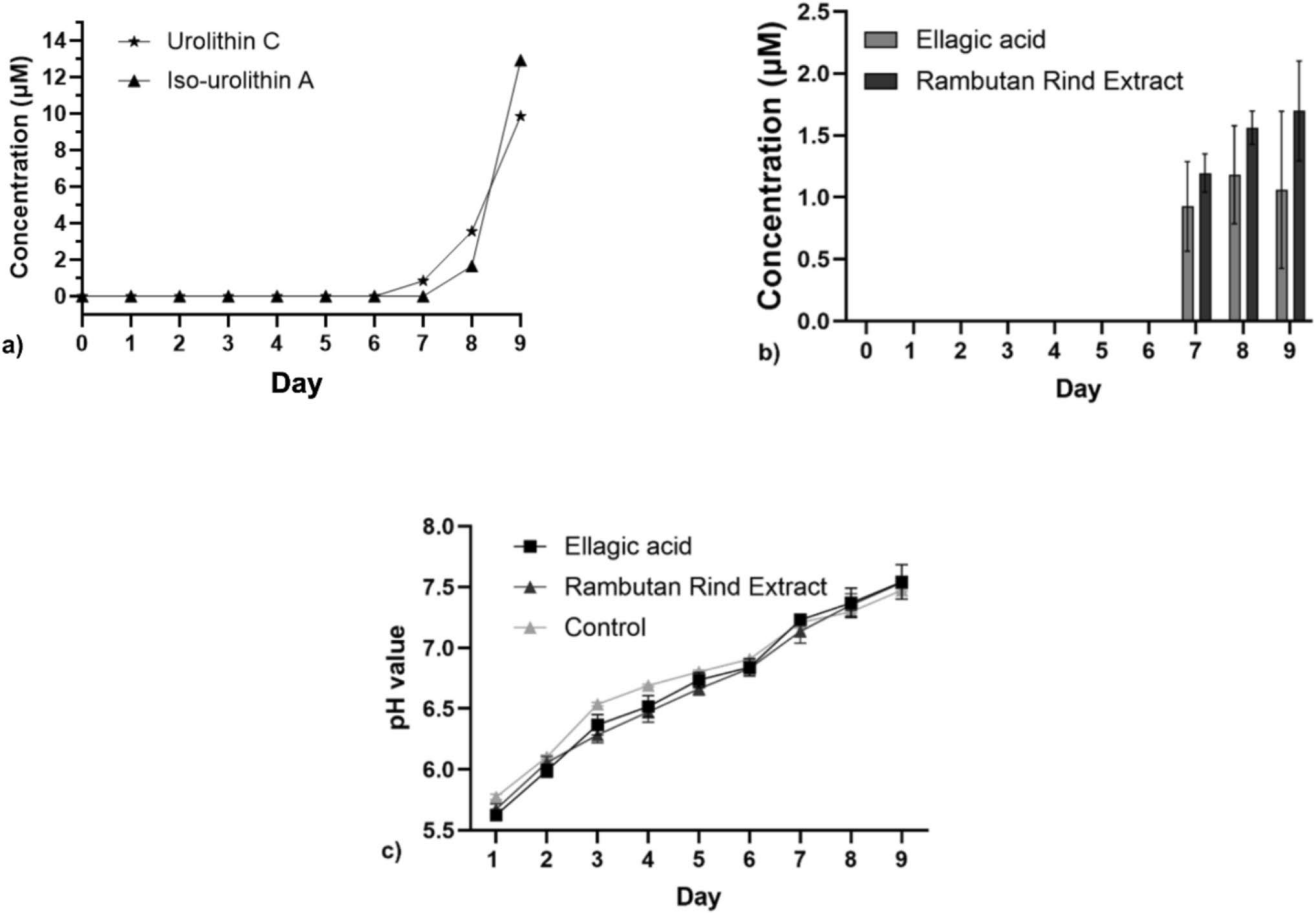
Time course of **a** urolithin production using the Sprague-Dawley rats’ faecal matter and hydrolysed rambutan rind extract in broth media at 113 µM EAE. Time course of **b** isourolithin A production by the mixed bacterial culture in anaerobic basal broth between rambutan rind extract and ellagic acid and **c** pH changes by the mixed bacterial culture in anaerobic basal broth between rambutan rinds extract and ellagic acid at 20 µM. Note: Data represent mean with ± SD of replicate cultures (*n* = 3)

For the ex vivo biotransformation study, ABB supplemented with hydrolysed RRE (113 µM EAE) was inoculated with rat faecal matter and incubated under anaerobic conditions. Fermentation of hydrolysed RRE resulted in the sequential production of Uro C followed by IsoUro A, with maximum concentrations of 9.4 ± 0.6 µM and 12.5 ± 0.6 µM, respectively, detected by day 9 (Fig. 3a).

These findings indicate that microbial consortia derived from Sprague-Dawley rat faecal samples are capable of converting EA into downstream Uro metabolites under anaerobic fermentation conditions. The appearance of Uro C followed by IsoUro A over the incubation period is consistent with stepwise biotransformation of EA-derived substrates in mixed microbial cultures.

### Substrate conversion efficacy between ellagic acid and rambutan rind extract

Initial experiments compared the conversion efficacy of two substrates, EA and RRE, at equivalent concentrations (20 µM EAE), using a mixed bacterial culture previously shown to produce Uro C and IsoUro A under anaerobic conditions. Over the 9-day incubation period, no statistically significant differences were observed between EA-supplemented and RRE-supplemented cultures in terms of IsoUro A formation (Fig. 3b). Uro C concentrations were below the limit of detection under these conditions and were therefore not included in the comparative analysis (Fig. 3b).

IsoUro A was not detectable from day 1 to day 6 for either substrate. However, IsoUro A became quantifiable from day 7 onward, with concentrations increasing through day 9. Two-way repeated-measure ANOVA revealed no statistically significant interaction between substrate type and time over the 9-day fermentation period. Consistently, no significant differences were observed between substrates on individual Uro production days (day 7: *p* = 0.9729, 95%CI: [−2.101, 1.564]; day 8: *p* = 0.9082, 95%CI: [−2.623, 1.862]; day 9: *p* = 0.9023, 95%CI: [−3.351, 2.075]). Uro C was not included as its concentration is below the LOD.

A gradual increase in culture pH was observed for both substrates over the incubation period (Fig. 3c).

### Shotgun metagenomics data processing and functional annotation pipeline of EA supplemented Uro-producing gut microbiota

**Fig. 4:**
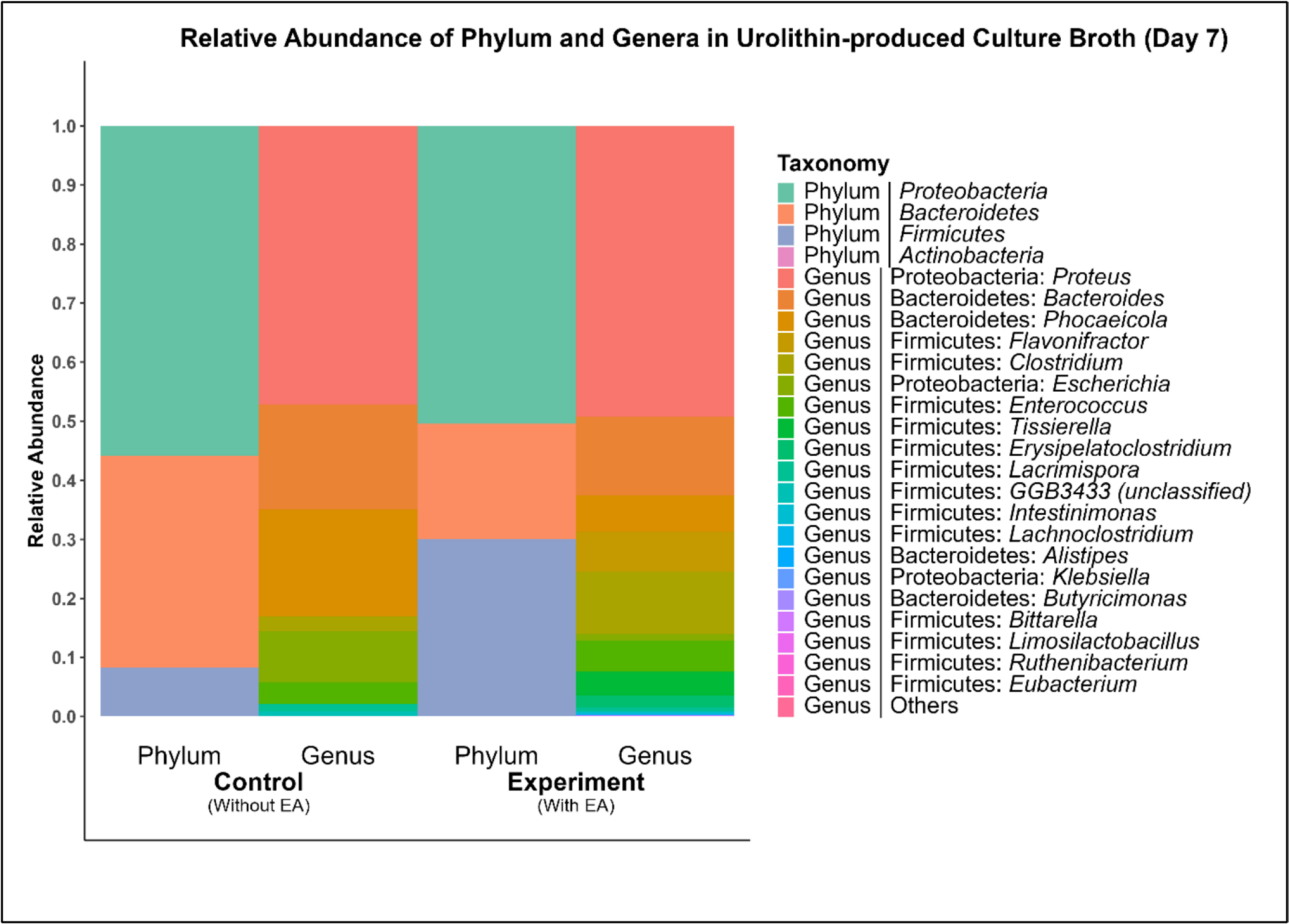
Relative abundance of phyla and genera in urolithin-producing culture broth at day 7. Note: Control group, without ellagic acid; experimental group, with ellagic acid (*n* = 1)

Shotgun metagenomic sequencing was used to characterise the taxonomic composition of microbial communities in EA-supplemented fermentation cultures and corresponding control cultures. At the phylum level, both groups were dominated by *Proteobacteria*, *Firmicutes*, and *Bacteroidetes*, which together accounted for the majority of the community composition (95.2%) (Fig. 4). In the EA-supplemented cultures, *Actinobacteria* were detected at very low relative abundance (< 0.01%), whereas this phylum was not detected in the control cultures.

Across both groups, *Proteobacteria* represented the most abundant phylum, accounting for approximately 51 to 56% of total relative abundance. While the overall community structure appeared broadly similar between groups, relative shifts were observed, with EA-supplemented cultures showing a higher proportion of *Firmicutes* (31.4%) and a lower proportion of *Bacteroidetes* (30.6%) compared with control cultures (*Firmicutes* 10%; *Bacteroidetes* 12.1%).

The Shannon α-diversity analysis indicated only minor differences in species richness and evenness indexes between EA-supplemented and control cultures at both the taxonomic (experimental 3.36; control 3.18) and predicted metabolic pathway levels (experimental 6.69; control 6.71). Together, these results indicate that overall microbial diversity was largely maintained under the conditions tested.

At the family level, EA-supplemented cultures were predominantly composed of *Morganellaceae*, *Bacteroidaceae*, *Clostridiaceae*, and *Oscillospiraceae*. In contrast, the control cultures were dominated by *Morganellaceae*, *Bacteroidaceae*, *Enterobacteriaceae*, *Enterococcaceae*, and *Clostridiaceae* (Supplementary Table 6).

These taxonomic profiles describe microbial composition associated with Uro-producing fermentation conditions and do not imply direct involvement of individual taxa in Uro biosynthesis.

**Fig. 5:**
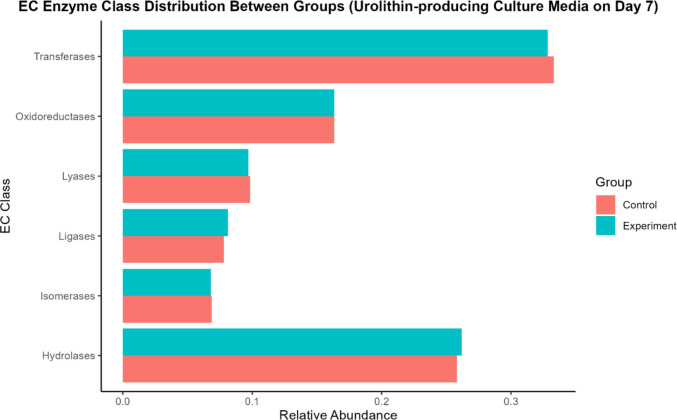
Enzyme commission distribution between groups (control vs experimental). Note: Control group, without ellagic acid; experimental group, with ellagic acid (*n* = 1)

**Fig. 6:**
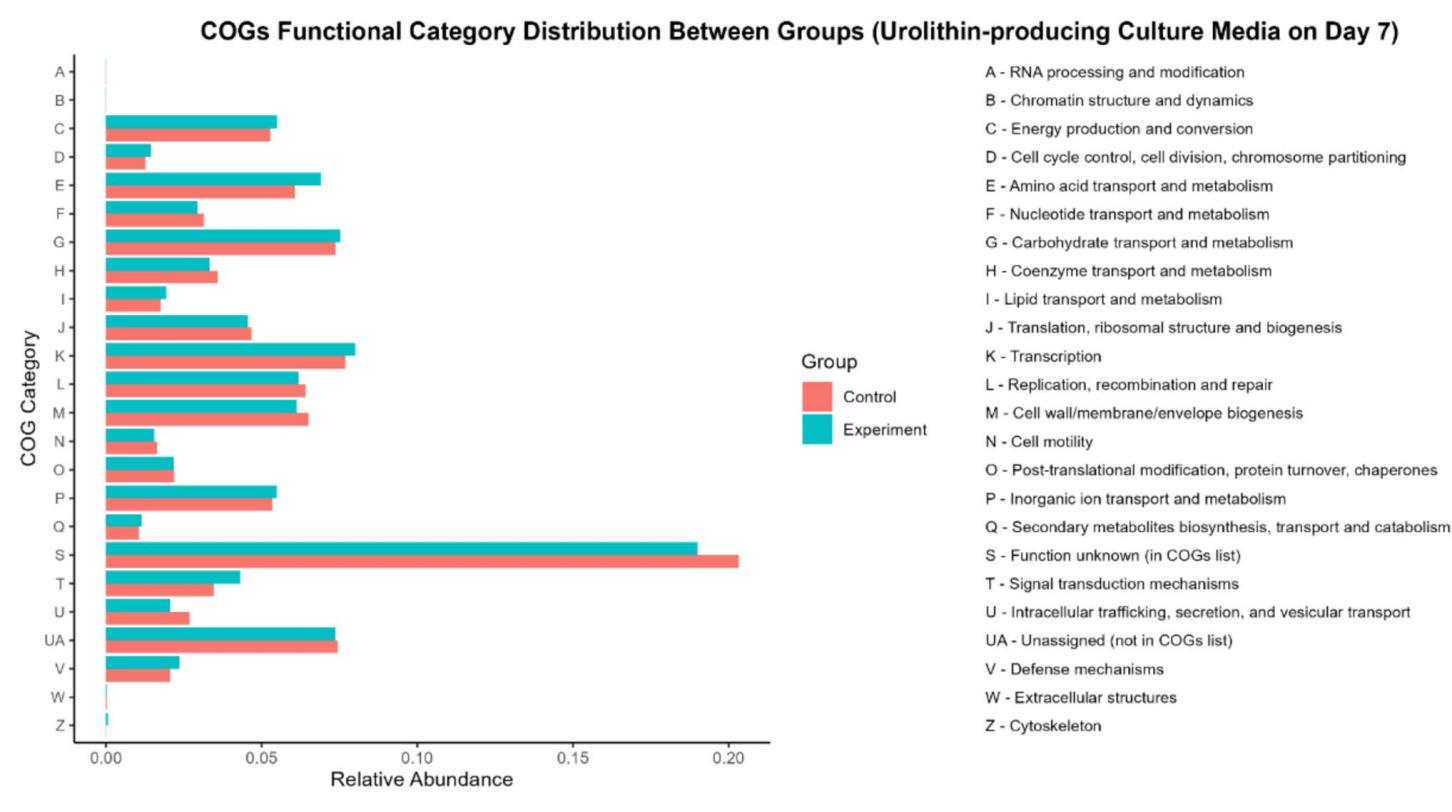
COG functional category distribution (control vs experimental group). Note: Control group, without ellagic acid; experimental group, with ellagic acid (*n* = 1)

**Fig. 7:**
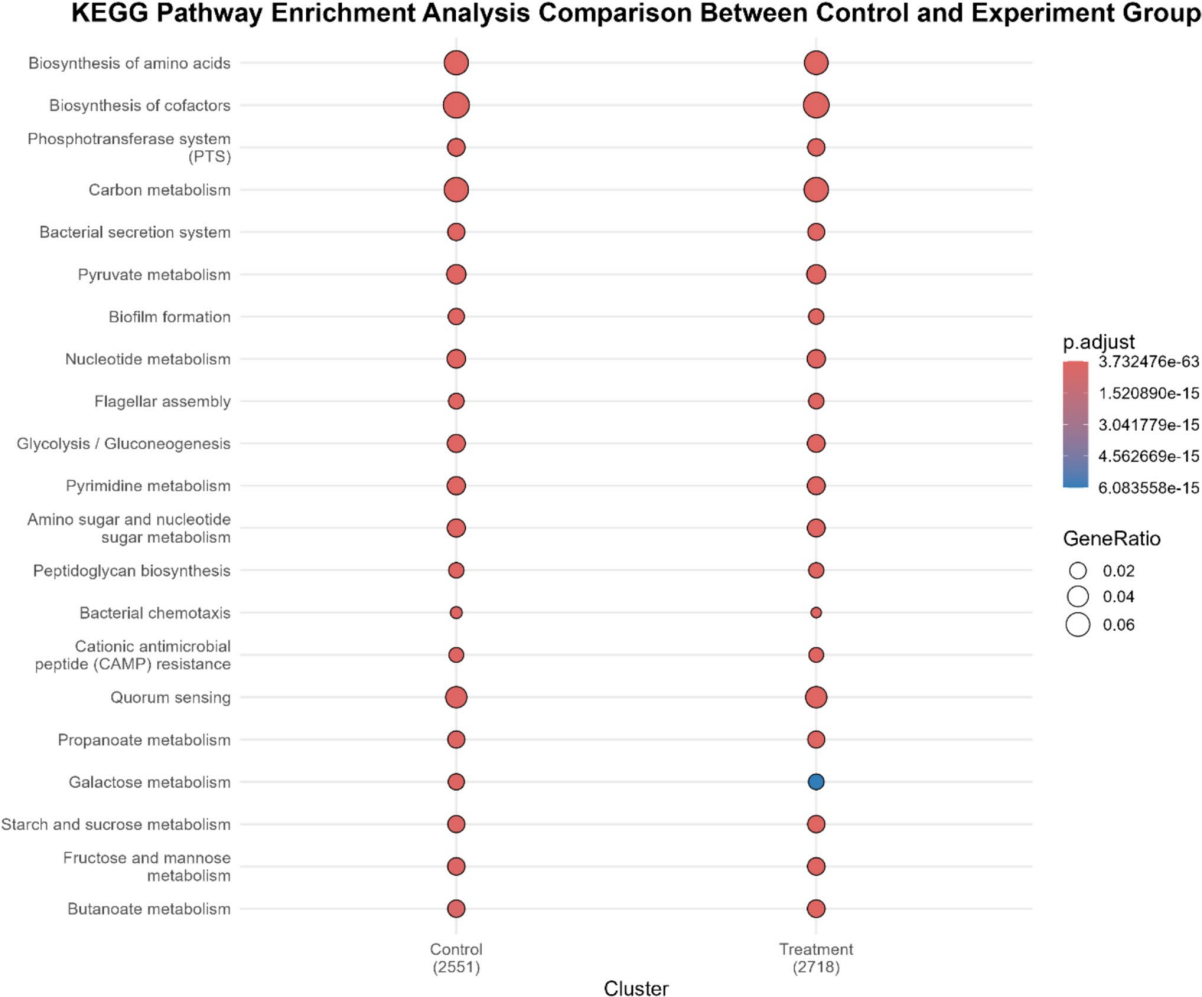
KEGG pathway enrichment analysis comparison (control vs experimental group). Control group, without ellagic acid; experimental group, with ellagic acid (*n* = 1)

Functional annotation using enzyme commission (EC) classification (Fig. 5), clusters of orthologous groups (COGs) (Fig. 6), and KEGG pathway enrichment analysis (Fig. 7) revealed that the overall distribution of predicted functional categories was broadly similar between EA-supplemented and control fermentation cultures, with only subtle variations observed. A substantial proportion of genes in both groups were assigned to COG category S (function unknown) reflecting the presence of uncharacterised functions within the metagenomic datasets.

Particularly, major COG functional categories, including M (cell wall/membrane/envelope biogenesis), C (energy production and conversion), G (carbohydrate transport and metabolism), and E (amino acid transport and metabolism) displayed comparable relative abundances between groups. These results suggest that the dominant predicted functional categories were largely conserved across the fermentation conditions examined, based on reference-based annotation.

As functional assignments are based on reference-dependent prediction, these profiles reflect inferred metabolic potential rather than confirmed functional activity.

### Comparative BLASTp and KEGG pathway analysis against *Ellagibacter isourolithinifaciens*

**Fig. 8:**
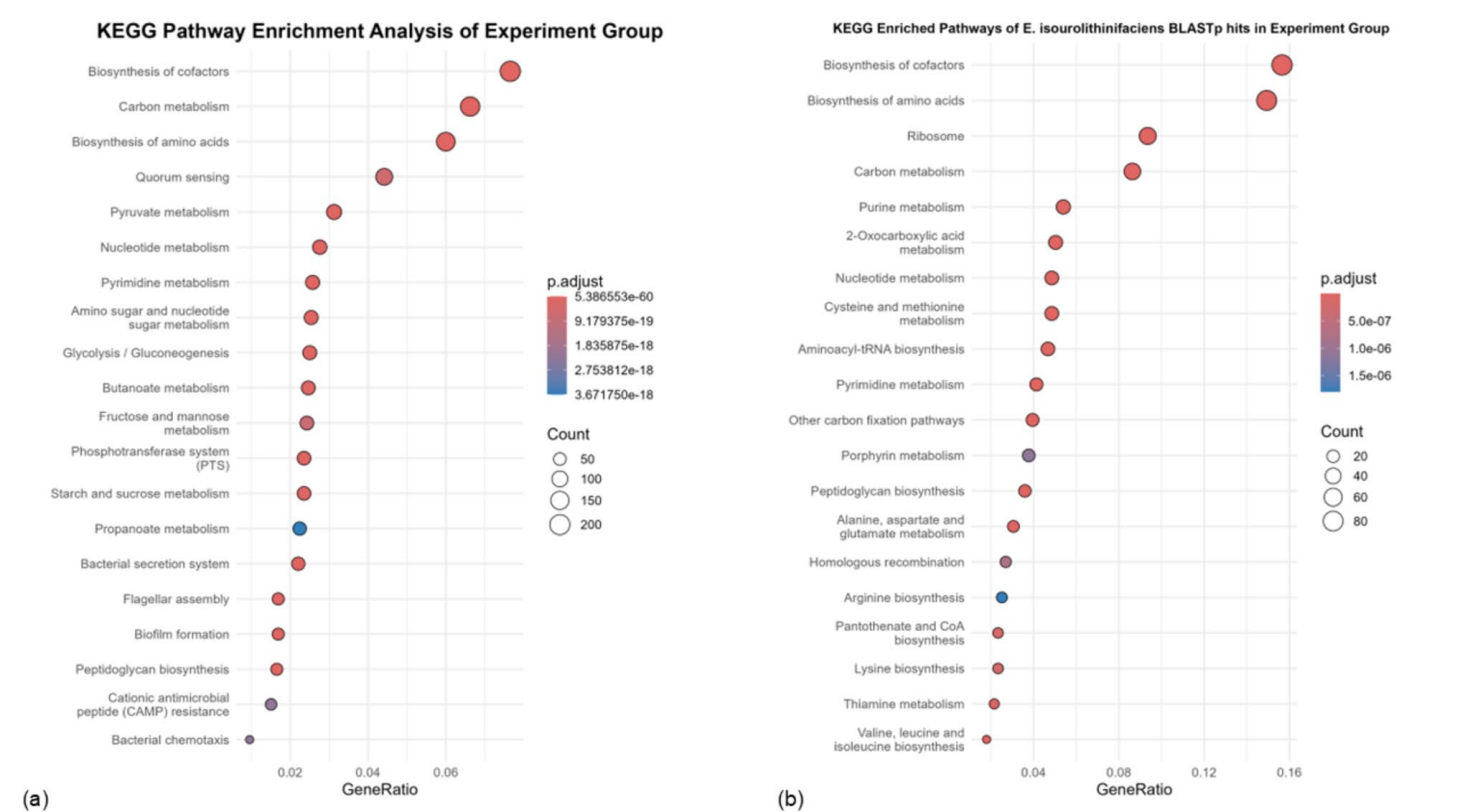
KEGG pathway enrichment analysis: **a** KEGG pathway enrichment of the experiment group and **b** KEGG-enriched pathways of *E. isourolithinifaciens DSM* 104140^T^ BLASTp hits in the experiment group

BLASTp-based comparison of predicted proteins from the experimental fermentation metagenome against the reference proteome of *E. isourolithinifaciens* DSM 104140^T^ revealed overlap in KEGG-annotated metabolic pathways, particularly in cofactor biosynthesis, amino acid biosynthesis, and carbon metabolism (Fig. 8). These shared pathway annotations reflect similarities in general metabolic functions rather than evidence of conserved Uro biosynthetic pathways.

Consistent with functional annotation results, no canonical Uro biosynthesis markers previously associated with EA degradation or Uro biosynthesis were detected, including punicalagin acyl hydrolase (EC 3.1.1.124), pyrocatechol dehydroxylase (EC 1.13.11.1), or the MetaCyc EA degradation pathway (PWY-7951).

The absence of these canonical markers may reflect either homology divergence or limitations of reference-based annotation for non-human fermentation.

### Identification of urolithin biosynthesis-related enzymes in the Sprague-Dawley gut microbiota

**Fig. 9:**
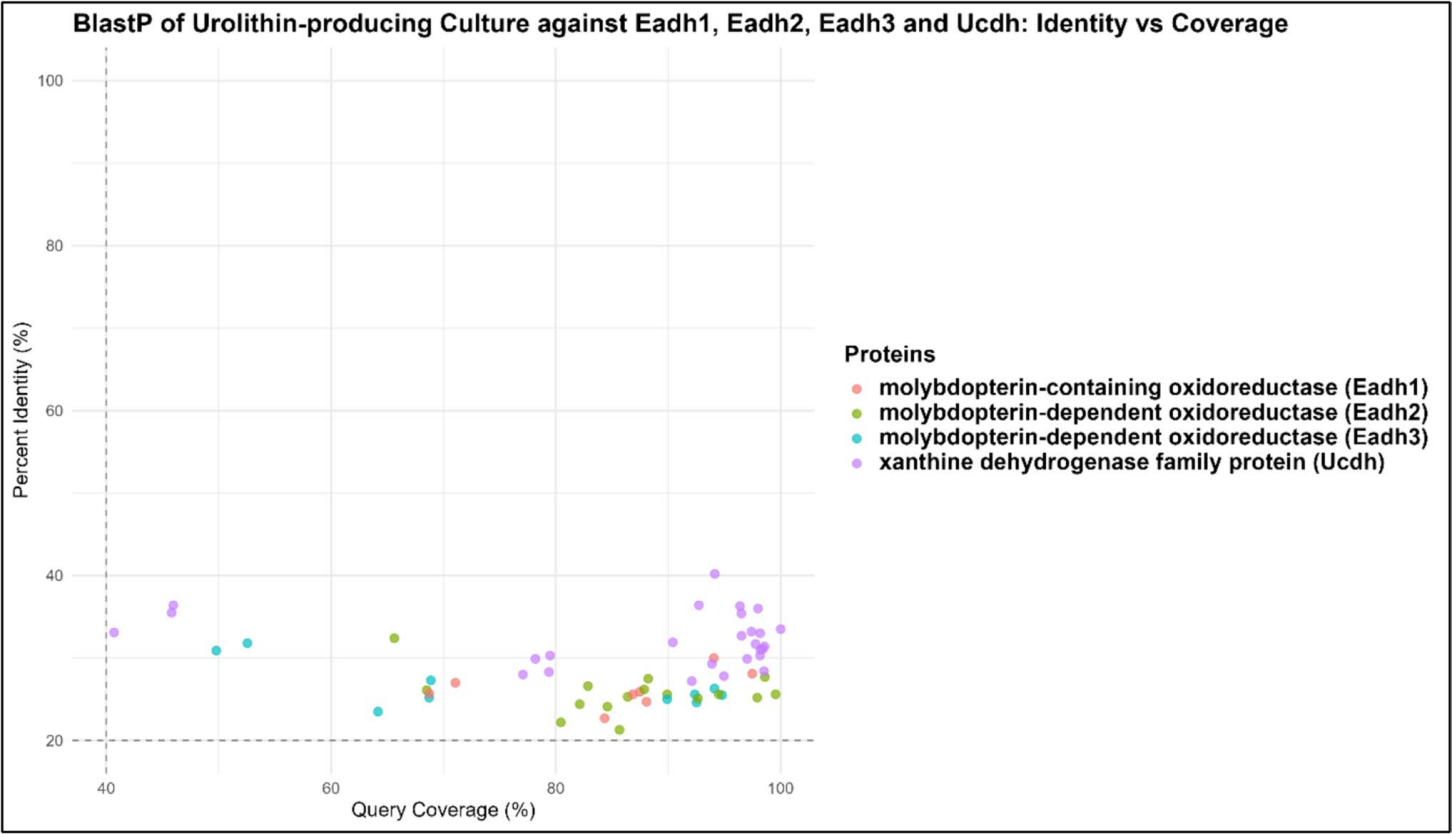
Identity vs coverage of urolithin-producing culture against Eadh1, Eadh2, Eadh3, and Ucdh

To assess the homology divergence and the presence of canonical Uro-producing genes, DIAMOND BLASTp searches were performed using a curated set of reference proteins associated with the Uro biosynthetic pathway (Supplementary Table 7). A total of 61 significant hits were identified and are illustrated in Fig. 9 (Supplementary Table 8). The percentage identity values observed across all enzyme categories were consistently low, ranging from 21.3 to 40.2%, suggesting the presence of functionally related but evolutionarily divergent homologues within the Uro-producing culture. Despite this sequence divergence, all hits returned statistically significant e-values (mean value of 1e^−12^ to 1e^−15^), with mean bitscores ranging from 124 ± 53.7 (Eadh3) to 213 ± 96.9 (Ucdh), where Ucdh exhibited the highest overall alignment scores, indicating these matches reflect genuine homology rather than stochastic alignments.

## Discussion

The histological assessment indicated that oral administration of RRE 115 mg/kg b.w. for 2 weeks did not result in observable histopathological alterations in the organs examined. These findings are consistent with previous reports describing the sub-chronic tolerability (12 weeks) of RRE at comparable doses (Moorthy et al. 2022). RRE is known to consist of a diverse range of ET and related polyphenolic compounds, including geraniin, corilagin, castalagin, and vescalagin (Palanisamy et al. 2011; Tingting et al. 2022) among others, which may collectively contribute to downstream microbial metabolism and the generation of bioactive metabolites. In parallel, faecal SCFA profiling revealed a reduction in isobutyric acid levels in RRE-fed rats. As isobutyric acid is a known marker associated with proteolytic fermentation in the gut (Zarling and Ruchim 1987), this observation is consistent with a shift in microbial metabolic output, although direct metabolic flux was not assessed (Duda-Chodak et al. 2015; Kilua et al. 2022).

Earlier studies on Uro fermentation have commonly used EA supplementation at concentrations between 10 and 30 µM (Gaya et al. 2018; Iglesias-Aguirre et al. 2023; Kim et al. 2024; Liu et al. 2022; Selma et al. 2014, 2017; Zhang et al., [Bibr CR27][Bibr CR28]). In the present study, fermentation was performed using hydrolysed RRE, which was pre-treated with HCl to release EA from ET (GE) naturally present in the rind (García-Villalba et al. 2015). Pre-hydrolysis of the RRE mimics the initial chemical and enzymatic transformations that occur during gastrointestinal digestion and increases the pool of bio-accessible EA available for microbial conversion. Fermentation with this higher EAE concentration (113 µM) yielded measurable levels of Uro C (9.4 ± 0.6 µM) and IsoUro A (12.5 ± 0.6 µM) by day 9. While direct comparison across studies is limited by differences in experimental design and microbial communities (Selma et al. 2017), these findings suggest that substrate concentration may influence the extent of detectable Uro formation under ex vivo conditions. The sequential appearance of Uro C (trihydroxy-Uro) followed by IsoUro A (dihydroxy-Uro) is consistent with previously described stepwise biotransformation patterns reported in mixed microbial cultures, although enzymatic steps were not directly examined in this study.

The use of RRE and hydrolysed RRE as substrates may also reduce reliance on purified ellagic acid and associated solvents. Excessive use of organic solvents for substrate solubilisation has been reported to influence microbial growth and metabolic activity in fermentation systems. In particular, high concentrations of PPG have been shown to inhibit the growth of certain bacterial species at concentrations above 10–12.5% (Mosiichuk et al. 2025). Although these observations were not made in gut-derived microbial communities, they highlight the potential for solvent-related effects to confound microbial fermentation outcomes. In addition, gradual increases in culture pH were observed during fermentation for both substrates. While near-neutral pH has been reported as favourable for microbial growth in Uro-producing cultures (Gaya et al. 2018; Zhang et al. 2023a), the observed pH changes likely reflect community-level metabolic activity rather than a prerequisite for Uro formation, particularly as the hydrolysed RRE was adjusted to neutral pH to mimic the environmental condition of the intestine prior to incubation.

A culture sample was collected for shotgun metagenomic analysis on day 7 as this corresponds to the first detectable appearance of Uro, representing the active phase of Uro production. At this stage, the microbial community and associated functional genes are most relevant to the bioconversion process. Furthermore*, E. isourolithinfaciens* has been reported to exhibit reduced production of IsoUro A after day 8 (Selma et al. 2017). Taxonomic profiling of Uro-producing fermentation cultures revealed that *Proteobacteria*, *Firmicutes*, and *Bacteroidetes* dominated community composition, with *Actinobacteria* detected only at very low relative abundance. This contrasts with previous reports in which Uro-producing isolates were predominantly recovered from *Actinobacteria*, particularly *Gordonibacter* and *Ellagibacter* species (Beltrán et al. 2018; Selma et al. 2014). The low abundance of *Actinobacteria* observed here suggests that Uro formation under the conditions tested may occur within microbial communities not dominated by previously characterised Uro-producing taxa. This observation aligns with broader evidence that polyphenol metabolism in the gut can be functionally redundant, with similar transformations potentially carried out by phylogenetically distinct microorganisms.

Functional annotation did not identify canonical enzymes or pathways previously associated with EA degradation and Uro biosynthesis, including punicalagin acyl hydrolase, pyrocatechol dehydroxylase, or the MetaCyc EA degradation pathway (Hua et al. 2024). This absence may reflect limitations of reference-based annotation pipelines when applied to non-human, fermentation-derived metagenomes, rather than the absence of functional capacity.

To further explore potential functional similarities, a BLASTp comparison between the protein-coding genes of *E. isourolithinifaciens* DSM 104140^T^, a characterised IsoUro A–producing strain, and the experimental fermentation metagenomes was conducted. This strain was selected as a reference due to its documented Uro production profile (Beltrán et al. 2018). Comparative analysis revealed overlap in several KEGG-enriched pathways, particularly those related to central carbon metabolism, amino acid biosynthesis, and cofactor biosynthesis, despite the absence of canonical Uro biosynthetic genes. These shared pathways likely reflect common metabolic frameworks that support microbial growth and polyphenol transformation rather than evidence of conserved or novel Uro biosynthetic pathways (Alqudah and Claesen 2024; Mosele et al. 2015). These observations highlight the possibility that Uro formation may be supported by alternative metabolic strategies that are not captured by currently annotated pathways, warranting further investigation using functional and expression-based approaches.

Given the absence of canonical markers, broader metabolic features were examined. NAD^+^ synthase was detected in multiple species-level genome bins present in both experimental and control fermentation cultures, including *Escherichia coli*, *Enterococcus faecalis*, and *Enterococcus avium*. As NAD^+^ synthase is ubiquitous and central to cellular metabolism, its presence alone cannot be interpreted as indicative of Uro-producing capacity. However, these taxa also encoded a range of oxidoreductases and hydrolytic enzymes, such as β-glucosidases and β-glucuronidases, which may enhance substrate accessibility by releasing aglycone forms of polyphenols. While these enzymes are not specific to Uro biosynthesis, their combined activity may contribute to a metabolic environment permissive for ET-derived transformations in mixed microbial communities.

Additionally, the present DIAMOND BLASTp analysis identified putative homologues corresponding to three catechol dehydroxylases (Eadh1, Eadh2, and Eadh3) and a xanthine oxidase family enzyme (Ucdh) within the Uro-producing culture. Ucdh exhibited consistently higher percentage identity (mean value of 32.2%) and bitscores (mean value of 213) compared to Eadh enzymes, suggesting it may be more evolutionarily conserved across gut microbial communities, or that xanthine oxidase family proteins are under stronger selective pressure due to broader metabolic roles. The observed percentage identity values fall within the “twilight zone”(Ben Boubaker et al. 2023), where relationships between sequence similarity and enzymatic function become ambiguous and structural or functional conservation cannot be inferred from the sequence alone. Such combinations of low sequence identity but substantial query coverage are frequently observed in metagenomic datasets when environmental homologues are evolutionarily distant from experimentally characterised reference enzymes. As the canonical Uro biosynthetic genes have been primarily characterised in human gut microbiota, the reduced sequence identity values observed here are not unexpected and may reflect host-specific or clade-specific divergence among Uro-pathway homologues in the animal-associated gut microbial communities. Although the majority of the BLASTp hits showed high query coverage, there remain a few hits from Eadh and Ucdh with query coverage below 50%. These findings suggest that only specific domains are conserved, which is consistent with distant homology, and further support the interpretation of functional divergence rather than orthologues’ conservation. Nonetheless, BLASTp-based homology inference alone is insufficient to confirm enzymatic function, and experimental validation such as heterologous expression or enzymatic assays would still be required to confirm the functional activity of these candidate genes.

Collectively, these findings suggest that Uro formation in the rat faecal-derived microbial consortium is unlikely to be mediated by direct orthologues of the currently characterised human gut enzymes. Instead, the observed sequence similarity patterns may reflect evolutionarily divergent homologues or alternative enzymatic systems capable of catalysing comparable dehydroxylation reactions. Moreover, the results demonstrate that RRE and hydrolysed RRE can serve as substrates supporting Uro formation in rat faecal-derived fermentation systems comparable to purified EA under the conditions tested, while highlighting functional associations within microbial consortia enriched under these conditions. These findings highlight the potential of agricultural fruit by-products as substrates for microbial polyphenol biotransformation and support their relevance in applied microbiology research.

Using an integrated fermentation, metabolite profiling, and shotgun metagenomic approach, this work characterised taxonomic and functional features associated with Uro-producing microbial consortia without attributing Uro biosynthesis to specific taxa or enzymes. The absence of canonical EA degradation genes, together with the presence of broadly shared metabolic pathways when compared with *E. isourolithinifaciens* DSM 104140^T^, suggests that Uro formation in this system may be supported by alternative or yet-uncharacterised metabolic strategies. Sequence similarity searches targeting Eadh1, Eadh2, Eadh3, and Ucdh identified only distant homologues (21.3 to 40.2% identity), consistent with functional analogues of the currently characterised Uro biosynthetic pathway. These homologues do not correspond to canonical pathway annotations, reinforcing the likelihood of pathway divergence rather than conservation.

The metagenomic findings presented in this study are descriptive and hypothesis-generating, providing a rationale for future research. These observations reinforce the limitations of reference-based functional annotation for non-human, fermentation-derived metagenomes and emphasise the value of discovery-driven approaches. Overall, this study establishes a methodological framework for investigating Uro biotransformation in non-human gut microbiomes and for exploring functional associations within complex microbial communities. Future studies integrating targeted cultivation, functional assays such as heterologous expression, isotope tracing, and meta-transcriptomic analyses will be required to definitively establish the enzymatic basis of Uro formation, validate functional activity, and identify the specific microbial contributors and enzymatic mechanisms involved.

## Supplementary Information

Below is the link to the electronic supplementary material.

ESM 1 (PDF 1.11 MB)

## Data Availability

WGS data have been deposited in SRA ([PRJNA1434035] (http://www.ncbi.nlm.nih.gov/bioproject/PRJNA1434035)). The original contributions presented in the study are included in the article, and further inquiries can be directed to the corresponding authors.

## Abbreviations

ABB: Anaerobic basal broth
ACN: Acetonitrile
CH_2_O_2_: Formic acid
COGs: Clusters of orthologous groups
dH_2_O: Distilled water
EA: Ellagic acid
EAE: Ellagic acid equivalent
EC: Enzyme commission
ET: Ellagitannin
FDA: Food and Drug Administration
GC: Gas chromatography
GE: Geraniin
GRAS: Generally Recognised as Safe
H&E: Harris’s haematoxylin and eosin
HCl: Hydrochloric acid
HPLC: High-performance liquid chromatography
IsoUro A: Isourolithin A
IS: Internal standard
MeOH: Methanol
MAGs: Metagenome-assembled genomes
MWD: Multiple wavelength detector
PBS: Phosphate-buffered saline
PPG: Propylene glycol
RRE: Rambutan rind extract
Uro: Urolithins
