# Supplementary material for: Metagenomic insights into urolithin formation from rambutan rind extract by rat faecal-derived microbiome
Source: Appl Microbiol Biotechnol. 2026 Apr 27;110:171. doi: 10.1007/s00253-026-13841-x (PMC13236738; doi:10.1007/s00253-026-13841-x)
Supplement: Supplementary file 1 [file 253_2026_13841_MOESM1_ESM.pdf]

Metagenomic Insights into Urolithin Formation from Rambutan Rind Extract by Rat Faecal-derived Microbial Consortia

Wai-Kit Tow[1], Cindy Shuan Ju Teh[2], Chien Wei Ooi[4], Ronald F.S. Lee[1], Maalini Krishnasamy[5], Uma Devi Palanisamy[3*], and Usha Sundralingam[1*]

[1]School of Pharmacy, Monash University Malaysia, Jalan Lagoon Selatan, Bandar Sunway 47500, Subang Jaya, Selangor, Malaysia (W.K.-T.: wai.tow@monash.edu; U.S.: usha.sundralingam@monash.edu; R.F.S.-L.: ronald.lee@monash.edu)
[2]Department of Medical Microbiology, Faculty of Medicine, Universiti Malaya, Kuala Lumpur, Malaysia (C.S.J.-T.: cindysjteh@um.edu.my)
[3]Jeffrey Cheah School of Medicine and Health Sciences, Monash University Malaysia, Jalan Lagoon Selatan, Bandar Sunway 47500, Subang Jaya, Selangor, Malaysia (U.D.P.: umadevi.palanisamy@monash.edu)
[4]Department of Chemical Engineering, School of Engineering, Monash University Malaysia, Jalan Lagoon Selatan, Bandar Sunway 47500, Subang Jaya, Selangor, Malaysia (C.W.-O.: ooi.chien.wei@monash.edu)
[5]Thomson Hospital, Kota Damansara 47810, Petaling Jaya, Selangor, Malaysia (M.K.: maalinik@tmclife.com)

*Corresponding authors (usha.sundralingam@monash.edu; umadevi.palanisamy@monash.edu)

Supplementary Materials

Supplementary Figures

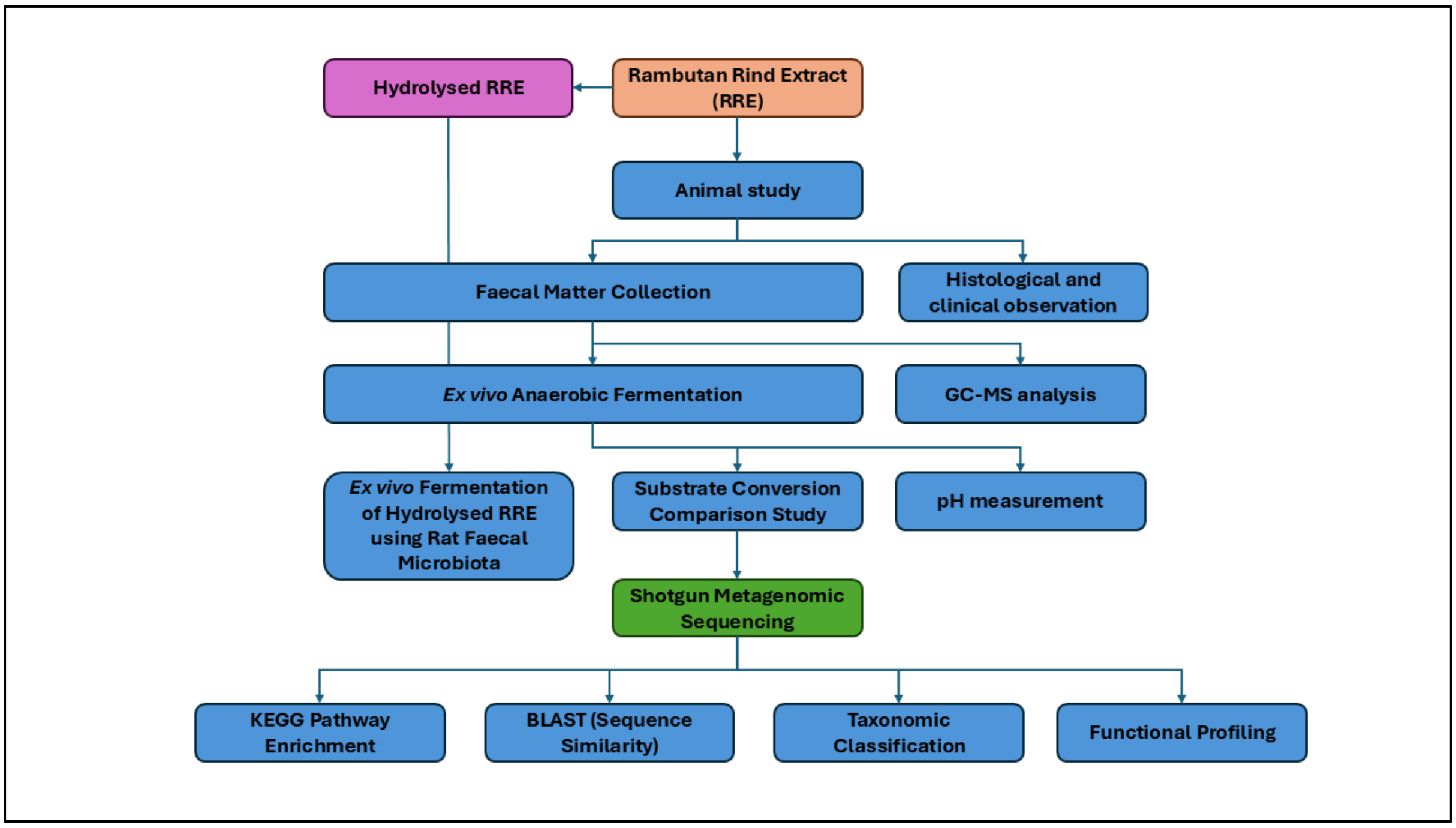

Supplementary Fig. 1 Workflow of the study.

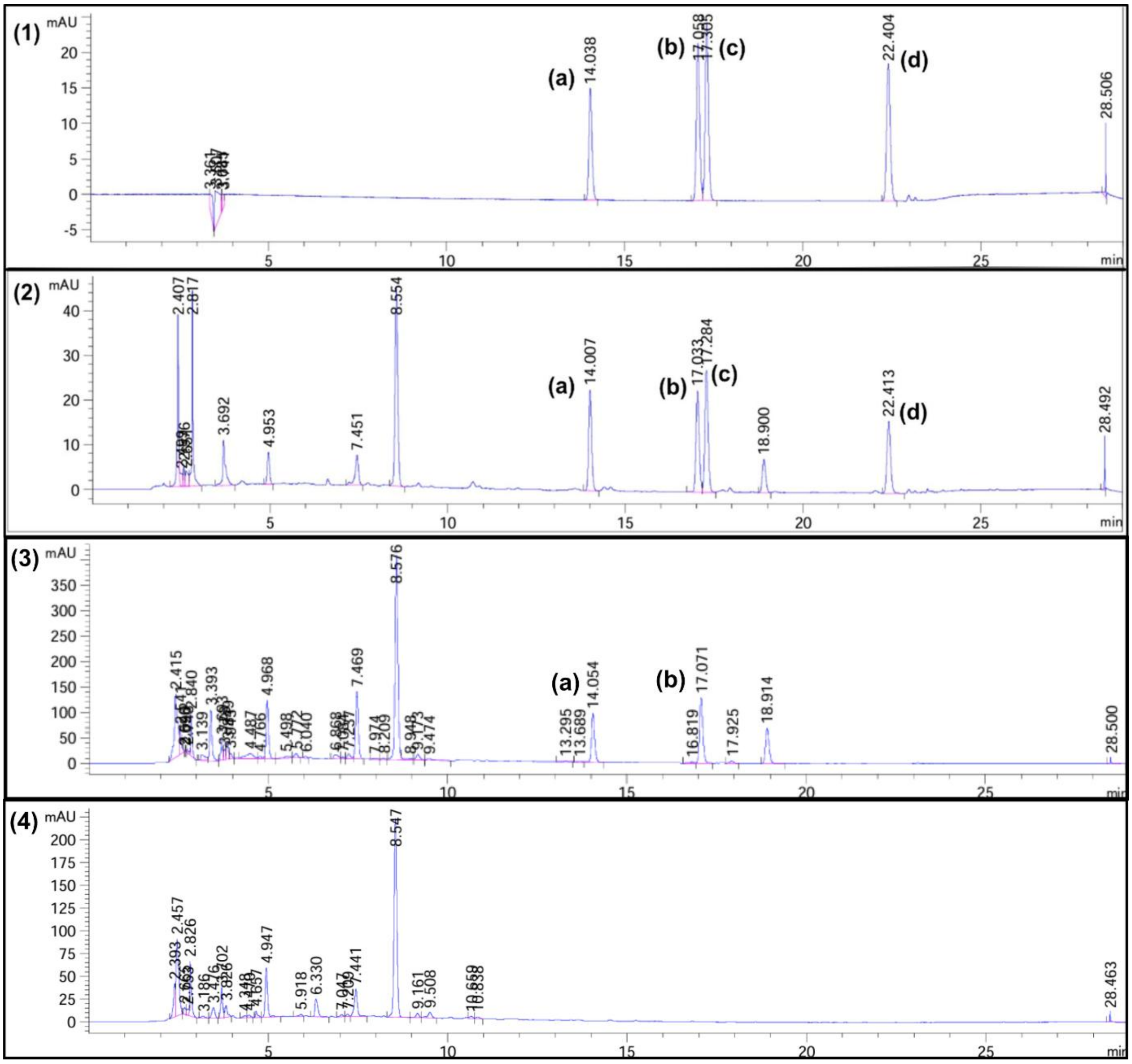

Supplementary Fig 2. Representative HPLC chromatogram showing the separation of urolithin standards in solvent, spiked broth samples, faecal matter inoculated broth media, and broth media (ABB). **(1)** Urolithins in solvent and **(2)** urolithins spiked in sample matrix at 12.5 µg/ml; **(3)** urolithins in Sprague Dawley rat's faecal matter inoculated broth media; **(4)** ABB media; **(a)** Uro C, **(b)** IsoUro A, **(c)** Uro A, and **(d)** Uro B.

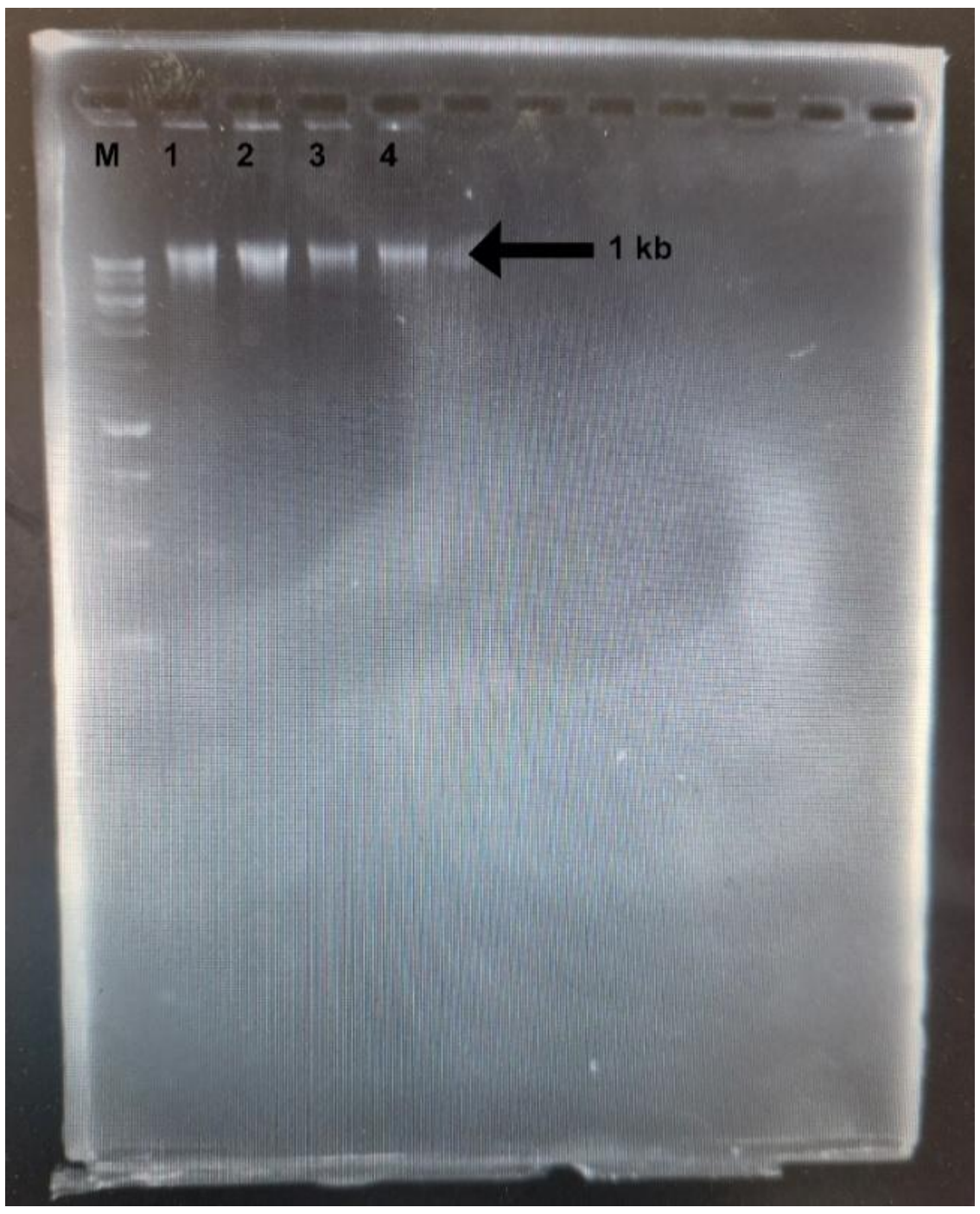

Supplementary Fig. 3 Agarose gel electrophoresis of control and experimental mixed bacterial culture genomic DNA.

Note: Lanes: M – 1 kb DNA ladder; 1 and 2 - EA supplemented fermentation cultures; 3 and 4 – Control cultures.

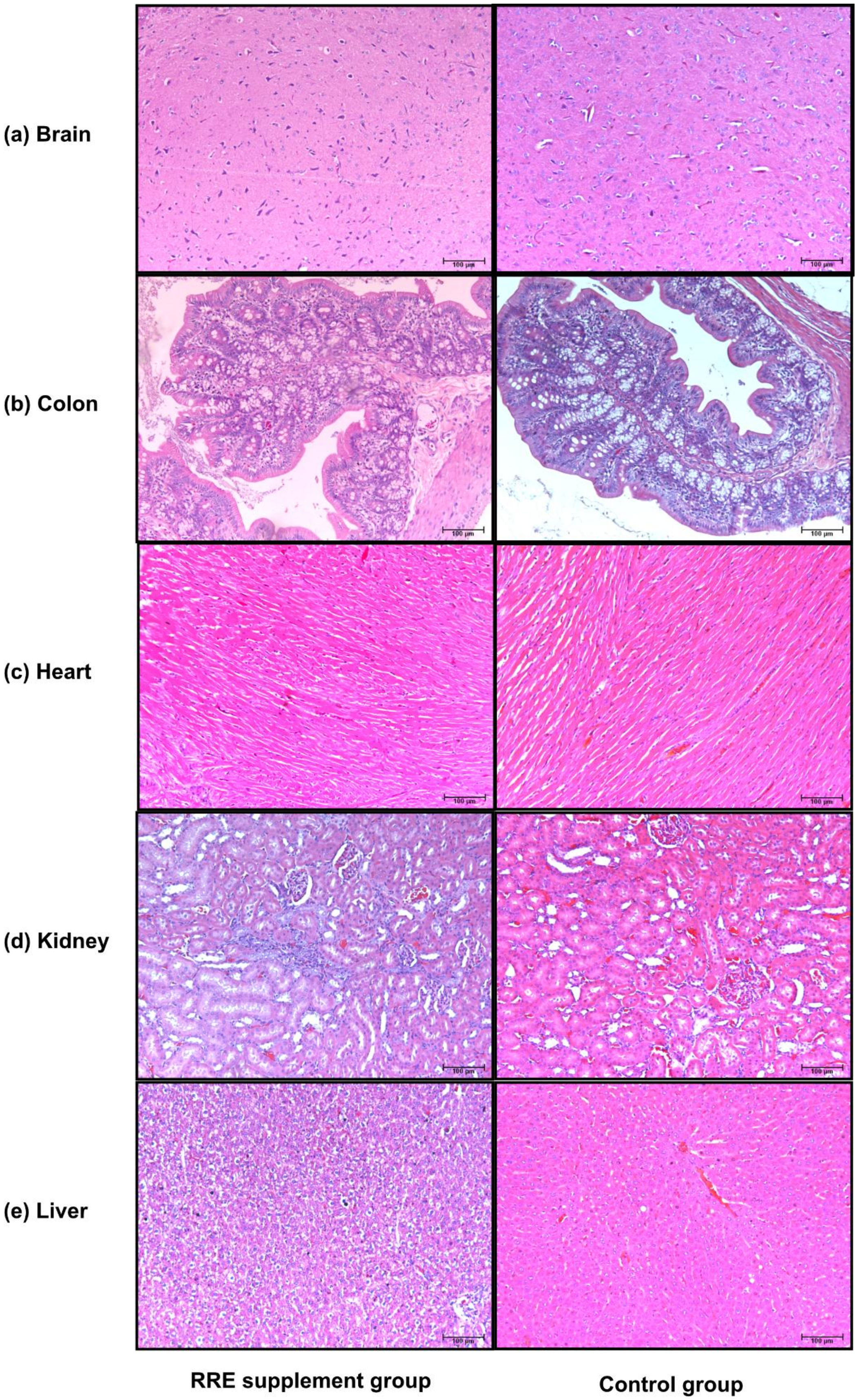

Supplementary Fig. 4 Representative photomicrographs (H&E, 10x) of (a) brain, (b) colon, (c) heart, (d) kidney, and (e) liver of control and RRE-treated Sprague Dawley rats (115 mg/kg b.w.) after 14 days of treatment (n = 5 per group)

Supplementary Table

Supplementary Table 1. Chromatographic conditions for urolithins quantification.

**HPLC Parameter**	**Details**		
Column	Luna Reversed Phase C18(2) (Phenomenex, CA, USA), 250 × 4.6 mm, 5µm particle sizes		
Flow rate	1.0 ml/min		
UV wavelength (λ)	305 nm		
Column temperature	24°C		
Mobile phase (A)	$H_2O:CH_2O_2$ (99.9:0.1; v/v)		
Mobile phase (B)	ACN		
Elution type	Gradient		
Total runtime	29 min		
Elution condition	Time (min)	A (%)	B (%)
	0	90	10
	20	50	50
	21	5	95
	25	0	100
	29	90	10
Post-run	12 min		

Supplementary Table 2. Statistical parameters of Uro calibration curve for HPLC-MWD.

**Compounds**	**Urolithin A**	**Urolithin B**	**Urolithin C**	**Iso-urolithin A**
Retention time (t_R)	17.29±0.02	22.39±0.02	14.02±0.01	17.04±0.01
%RSD t_R	0.11	0.09	0.12	0.05
Linearity range[a]	3.125 – 100			
Regression equation	y = 13036x-2.111	y = 11387x-1.626	y = 7971x-3.191	y = 11500x-2.113
Correlation coefficient (r^2)	1	1	1	1
LOD[b]	0.13 (0.60)	0.14 (0.69)	0.27 (1.12)	0.15 (0.68)
LOQ[b]	0.41 (1.81)	0.44 (2.10)	0.83 (3.41)	0.47 (2.07)
Standard error	5.43E-01	6.64E-01	5.08E-01	5.41E-01
F	8.13E+05	6.86E+05	5.58E+05	7.20E+05
SS (residual)	3.6E+00	5.22E+00	8.92E+00	5.98E+00
MS (residual)	9.05E-01	1.04E+00	1.78E+00	1.19E+00
SS (regression)	1.16E+06	9.98E+05	4.88E+05	1.01E+06
MS (regression)	1.16E+06	9.98E+05	4.88E+05	1.01E+06
Lower 95% (Slope)	1.30E+04	1.13E+04	7.94E+03	1.14E+04
Upper 95% (Slope)	1.30E+04	1.14E+04	7.99E+03	1.15E+04
Half-width	30.5	29.0	22.5	28.5
% Relative uncertainty (u_r)[c]	0.23	0.25	0.28	0.24

Supplementary Table 3. Inter- and Intra-day statistics of urolithin quantification using HPLC-MWD.

Compounds	Concentration (μg/ml)	Intra-day Accuracy (%±SD)		Inter-day Precision (%RSD)	
		Day 1		Day 2	Day 3
**Urolithin A**	100	99.46±0.41	0.42	0.19	0.40
	50	98.17±0.19	0.19	0.56	0.73
	25	98.69±0.36	0.37	0.08	0.45
**Urolithin B**	100	99.55±0.32	0.32	0.15	0.42
	50	98.34±0.24	0.24	0.55	0.75
	25	98.82±0.18	0.18	0.13	0.52
**Urolithin C**	100	99.39±0.26	0.26	1.11	0.75
	50	99.89±0.02	0.02	0.95	1.27
	25	93.45±3.45	3.70	3.33	4.23
**Iso-urolithin A**	100	99.63±0.45	0.45	0.19	0.38
	50	98.48±0.24	0.24	0.52	0.79
	25	99.60±0.31	0.31	0.03	0.45

Supplementary Table 4. Separation resolution (R_s) between urolithin A and iso-urolithin A.

Concentration	R_s±SD
0.1	2.50±0.02
0.05	2.52±0.00
0.025	2.53±0.03
0.0125	2.55±0.02
0.00625	2.58±0.03
0.003125	2.66±0.10

Supplementary Table 5. Matrix effects compared using the calibration curve slope between standards spiked in broth and prepared in solvent.

Compound	Sample matrix	Regression equation y = (m±SD_m)x-(c±SD_c)	Slope ratio of broth to solvent	*p*-value
**Uro A**	Broth	y = (13663±74.46)x-(4.18±3.74)	1.048±0.008	0.949
	Solvent	y = (13036±52.58)x-(2.11±0.62)		
**Uro B**	Broth	y = (9107±47.25)x-(1.75±2.53)	0.799±0.005	0.741
	Solvent	y = (11387±50.27)x-(1.62±0.57)		
**Uro C**	Broth	y = (9483±61.44)x-(7.99±4.02)	1.189±0.008	0.752
	Solvent	y = (7971±30.81)x-(3.19±0.47)		
**Iso-uro A**	Broth	y = (11148±60.63)x-(2.57±1.05)	0.969±0.008	0.970
	Solvent	y = (11500±47.93)x-(2.11±0.59)		

Note: p-value: $p < 0.05$ as statistically significant; y, peak area; x, concentration; m, slope; SD_m, standard deviation of slope; SD_c, standard deviation of intercept.

Supplementary Table 6. Top 10 hits of the most abundant families in EA-supplemented and control cultures.

No.	With EA		Without EA	
	**Family**	**Relative abundance**	**Family**	**Relative abundance**
1	*Morganellaceae*	49.3	*Morganellaceae*	47.2
2	*Bacteroidaceae*	19.4	*Bacteroidaceae*	35.8
3	*Clostridiaceae*	10.6	*Enterobacteriaceae*	8.64
4	*Oscillospiraceae*	6.88	*Enterococcaceae*	3.78
5	*Enterococcaceae*	5.24	*Clostridiaceae*	2.48
6	*Tissierellaceae*	4.02	*Lachnospiraceae*	1.98
7	*Erysipelotrichaceae*	2.03	*Lactobacillaceae*	0.041
8	*Enterobacteriaceae*	1.14	*Oscillospiraceae*	0.040
9	*Lachnospiraceae*	0.862		
10	*Eubacteriales*	0.367		

Supplementary Table 7. Source organisms, gene symbol, and accession number of urolithin-producing associated proteins.

Protein Name	Gene Symbol	Accession Number	Source Organism
Molybdopterin-containing oxidoreductase	Eadh1	WP_114602618.1	Gordonibacter sp. 28C
Molybdopterin-dependent	Eadh2	WP_114601864.1	Gordonibacter sp. 28C
Molybdopterin-dependent oxidoreductase	Eadh3	WP_158048609.1	Ellagibacter isourolithinifaciens DSM 104140
Xanthine dehydrogenase family protein	Ucdh	WP_002569573.1	Enterocloster bolteae DSM 15670

Supplementary Table 8. Number of hits, mean percentage of identity and coverage of urolithin-producing associated proteins.

Protein	Hits	Percentage of identity (mean±SD)	Coverage (mean±SD)	Bitscore (mean±SD)	e-value (mean)
molybdopterin-containing oxidoreductase (Eadh1)	8	26.2±2.20	84.8±10.1	156±75.5	$1.99e^{-12}$
molybdopterin-dependent oxidoreductase (Eadh2)	16	25.7±2.45	86.6±9.65	145±50.2	$5.83e^{-15}$
molybdopterin-dependent oxidoreductase (Eadh3)	10	26.6±2.72	76.8±17.9	124±53.7	$2.52e^{-14}$
xanthine dehydrogenase family protein (Ucdh)	27	32.2±3.27	87.9±17.2	213±96.9	$2.67e^{-14}$
